# On the Relevance of Assumptions Associated with Classical Factor Analytic Approaches^†^

**DOI:** 10.3389/fpsyg.2013.00109

**Published:** 2013-03-27

**Authors:** Daniel Kasper, Ali Ünlü

**Affiliations:** ^1^Chair for Methods in Empirical Educational Research, TUM School of Education and Centre for International Student Assessment, Technische Universität MünchenMunich, Germany

**Keywords:** factor analysis, latent variable model, normality assumption, factorial structure, criterion-referenced test, large scale educational assessment, Programme for International Student Assessment, Progress in International Reading Literacy Study

## Abstract

A personal trait, for example a person’s cognitive ability, represents a theoretical concept postulated to explain behavior. Interesting constructs are latent, that is, they cannot be observed. Latent variable modeling constitutes a methodology to deal with hypothetical constructs. Constructs are modeled as random variables and become components of a statistical model. As random variables, they possess a probability distribution in the population of reference. In applications, this distribution is typically assumed to be the normal distribution. The normality assumption may be reasonable in many cases, but there are situations where it cannot be justified. For example, this is true for criterion-referenced tests or for background characteristics of students in large scale assessment studies. Nevertheless, the normal procedures in combination with the classical factor analytic methods are frequently pursued, despite the effects of violating this “implicit” assumption are not clear in general. In a simulation study, we investigate whether classical factor analytic approaches can be instrumental in estimating the factorial structure and properties of the population distribution of a latent personal trait from educational test data, when violations of classical assumptions as the aforementioned are present. The results indicate that having a latent non-normal distribution clearly affects the estimation of the distribution of the factor scores and properties thereof. Thus, when the population distribution of a personal trait is assumed to be non-symmetric, we recommend avoiding those factor analytic approaches for estimation of a person’s factor score, even though the number of extracted factors and the estimated loading matrix may not be strongly affected. An application to the Progress in International Reading Literacy Study (PIRLS) is given. Comments on possible implications for the Programme for International Student Assessment (PISA) complete the presentation.

## 1 Introduction

Educational research is concerned with the study of processes of learning and teaching. Typically, the investigated processes are not observable, and to unveil these, manifest human behavior in test situations is recorded. According to Lienert and Raatz (1998, p. 1) “a test […] is a routine procedure for the investigation of one or more empirically definable personality traits” (translated by the authors), and to satisfy a minimum of quality criteria, a test is required to be objective, reliable, and valid.

In this paper we deal with factor analytic methods for assessing construct validity of a test, in the sense of its factorial validity (e.g., Cronbach and Meehl, 1955; Lienert and Raatz, 1998). Factorial validity refers to the factorial structure of the test, that is, to the number (and interpretation) of underlying factors, the correlation structure among the factors, and the correlations of each test item with the factors. There are a number of latent variable models that may be used to analyze the factorial structure of a test – for generalized latent variable modeling covering a plethora of models as special cases of a much broader framework, see Bartholomew et al. (2011) and Skrondal and Rabe-Hesketh (2004). This paper focuses on classical factor analytic approaches, and it examines how accurately different methods of classical factor analysis can estimate the factorial structure of test data, if assumptions associated with the classical approaches are not satisfied. The methods of classical factor analysis will include principal component analysis (PCA; Pearson, 1901; Hotelling, 1933a,b; Kelley, 1935), exploratory factor analysis (EFA; Spearman, 1904; Burt, 1909; Thurstone, 1931, 1965), and principal axis analysis (PAA; Thurstone, 1931, 1965). More recent works on factor analysis and related methods are Harman (1976), McDonald (1985), Cudeck and MacCallum (2007), and Mulaik (2009). Further references, to more specific topics in factor analysis, are given below, later in the text.^1^

A second objective of this paper is to examine the scope of these classical methods for estimating the probability distribution of latent ability values or properties thereof postulated in a population under investigation, especially when this distribution is skewed (and not normal). In applied educational contexts, for instance, that is not seldom the practice. Therefore a critical evaluation of this usage of classical factor analytic methods for estimating distributional properties of ability is important, as we do present with our simulation study in this paper, in which metric scale (i.e., at least interval scale; not dichotomous) items are used.

The results of the simulation study indicate that having a non-normal distribution for latent variables does not strongly affect the number of extracted factors and the estimation of the loading matrix. However, as shown in this paper, it clearly affects the estimation of the latent factor score distribution and properties thereof (e.g., skewness).

More precisely, the “estimation accuracy” for factorial structure of these models is shown to be worse when the assumption of interval-scaled data is not met or item statistics are skewed. This corroborates related findings published in other works, which we briefly review later in this paper. More importantly, the empirical distribution of estimated latent ability values is biased compared to the true distribution (i.e., estimates deviate from the true values) when population abilities are skewly distributed. It seems therefore that classical factor analytic procedures, even though they are performed with metric (instead of non-metric) scale indicator variables, are not appropriate approaches to ability estimation when skewly distributed population ability values are to be estimated.

Why should that be of interest? In large scale assessment studies such as the Programme for International Student Assessment (PISA)^2^ latent person-related background (conditioning) variables such as sex or socioeconomic status are obtained as well by principal component analysis, and that “covariate” information is part of the PISA procedure that assigns to students their literacy or plausible values (OECD, 2012; see also Section 3.1 in the present paper). Now, if it is assumed that the distribution of latent background information conducted through questionnaires at the students, schools, or parents levels (the true latent variable distribution) is skewed, based on the simulation study of this paper we can expect that the empirical distribution of estimated background information (the “empirical” distribution of the calculated component scores) is biased compared to the true distribution (and is most likely skewed as well). In other words, estimated background values do deviate from their corresponding true values they ought to approximate, and so the inferred students’ plausible values may be biased. Further research is necessary in order to investigate the effects and possible implications of potentially biased estimates of latent background information on students’ assigned literacy values and competence levels, based on which the PISA rankings of OECD countries are reported. For an analysis of empirical large scale assessment (Progress in International Reading Literacy Study; PIRLS) data, see Section 6.

The paper is structured as follows. We introduce the considered classical factor analysis models in Section 2 and discuss the relevance of the assumptions associated with these models in Section 3. We describe the simulation study in Section 4 and present the results of it in Section 5. We give an empirical data analysis example in Section 6. In Section 7, we conclude with a summary of the main findings and an outlook on possible implications and further research.

## 2 Classical Factor Analysis Methods

We consider the method of principal component analysis on the one hand, and the method of exploratory factor and principal axis analysis on the other. At this point recall Footnote 1, where we clarified that, strictly speaking, principal component analysis is not factor analysis and that principal axis analysis is a specific method for estimating the exploratory factor analysis model. Despite this, for the sake of simplicity and for our purposes and analyses, we call these approaches collectively factor analysis/analytic methods or even models. For a more detailed discussion of these methods, see Bartholomew et al. (2011).

Our study shows, amongst others, that the purely computational dimensionality reduction method PCA performs surprisingly well, as compared to the results obtained based on the latent variable models EFA and PAA. This is important, because applied researchers often use PCA in situations where factor analysis more closely matches their purpose of analysis. In general, such computational procedures as PCA are easy to use. Moreover, the comparison of EFA (based on ML) with PAA (eigenstructure of the reduced correlation matrix based on communality estimates) in this paper represents an evaluation of different estimation procedures for the classical factor analysis model. This comparison of the two estimation procedures seems to be justified and interesting, as the (manifest) normality assumption in the observed indicators for the ML procedure is violated, both in the simulation study and empirical large scale assessment PIRLS application. At this point, see also Footnote 1.

### 2.1 Principal component analysis

The model of principal component analysis (PCA) is

$$\text{Z}={\text{FL}}^{\prime },$$

where ***Z*** is a *n* × *p* matrix of standardized test results of *n* persons on *p* items, ***F*** is a *n* × *p* matrix of *p* principal components (“factors”), and ***L*** is a *p* × *p* loading matrix.^3^ In the estimation (computation) procedure ***F*** and ***L*** are determined as ***F*** = ***ZC*Λ**^−1/2^ and ***L*** = ***C*Λ**^1/2^ with a *p* × *p* matrix **Λ** = diag{λ_1_, …, λ*_p_*}, where λ*_l_* are the eigenvalues of the empirical correlation matrix ***R*** = ***Z′Z***, and with a *p* × *p* matrix ***C*** = (***c*_1_**, …, ***c_p_***) of corresponding eigenvectors ***c_l_***.

In principal component analysis we assume that ***Z*** ∈ ℝ*^n×*p*^*, ***F*** ∈ ℝ*^n×*p*^*, and ***L*** ∈ ℝ*^n×*p*^* and that empirical moments of the manifest variables exist such that, for any manifest variable *j* = 1, …, *p*, its empirical variance is not zero ($s_{j}^{2}\neq 0$). Moreover we assume that rk(***Z***) = rk(***R***) = *p* (rk, the matrix rank) and that ***Z***, ***F***, and ***L*** are interval-scaled (at the least).

The relevance of the assumption of interval-scaled variables for classical factor analytic approaches is the subject matter of various research works, which we briefly discuss later in this paper.

### 2.2 Exploratory factor analysis

The model of exploratory factor analysis (EFA) is

y=μ+Lf+e,

where ***y*** is a *p* × 1 vector of responses on *p* items, **μ** is the *p* × 1 vector of means of the *p* items, ***L*** is a *p* × *k* matrix of factor loadings, ***f*** is a *k* × 1 vector of ability values (of factor scores) on *k* latent continua (on factors), and ***e*** is a *p* × 1 vector subsuming remaining item specific effects or measurement errors.

In exploratory factor analysis, we assume that

$$\begin{array}{llll} \text{y}\in {\mathbb{R}}^{p\times 1}, & \;\mu \in {\mathbb{R}}^{p\times 1},\text{L}\in {\mathbb{R}}^{p\times k},\text{f}\in {\mathbb{R}}^{k\times 1},\;\text{and}\;\text{e}\in {\mathbb{R}}^{p\times 1}, & & \\ \text{y,}\;\mu ,\;\text{L, f,} & \;\text{and e are interval - scaled (at the least),} & & \\ \text{E}\left(\text{f}\right) & =\text{0}, & & \\ \text{E}\left(\text{e}\right) & =\text{0}, & & \\ \text{cov}\left(\text{e},\text{e}\right) & =\text{E}\left({\text{ee}}^{\prime }\right)=\text{D}=\text{diag}\left\{v_{1},\ldots ,v_{p}\right\}, & & \\ \text{cov}\left(\text{f},\text{e}\right) & =\text{E}\left({\text{fe}}^{\prime }\right)=\text{0}, & & \end{array}$$

where ν*_i_* are the variances of *e_i_* (*i* = 1, …, *p*). If the factors are not correlated, we call this the orthogonal factor model; otherwise it is called the oblique factor model. In this paper, we investigate the sensitivity of the classical factor analysis model against violated assumptions only for the orthogonal case (with cov(***f***, ***f*** ) = E(***ff′***) = ***I*** = diag{1, …, 1}).

Under this orthogonal factor model, **Σ** can be decomposed as follows:

$$\Sigma =\text{E}\left[\left(\text{y}-\mu \right){\left(\text{y}-\mu \right)}^{\prime }\right]=\text{E}\left[\left(\text{Lf}+\text{e}\right){\left(\text{Lf}+\text{e}\right)}^{\prime }\right]={\text{LL}}^{\prime }+\text{D}.$$

This decomposition is utilized by the methods of unweighted least squares (ULS), generalized least squares (GLS), or maximum likelihood (ML) for the estimation of ***L*** and ***D***. For ULS and GLS, the corresponding discrepancy function is minimized with respect to ***L*** and ***D*** (Browne, 1974). ML estimation is performed based on the partial derivatives of the logarithm of the Wishart (*W*) density function of the empirical covariance matrix ***S***, with (*n* − 1) ***S*** ∼ W (**Σ**, *n* − 1) (Jöreskog, 1967). After estimates for ***μ***, *k*, ***L***, and ***D*** are obtained, the vector ***f*** can be estimated by $\hat{f}={\left(L^{\prime }D^{-1}L\right)}^{-1}L^{\prime }D^{-1}(y-\mu ).$

When applying this exploratory factor analysis, ***y*** is typically assumed to be normally distributed, and hence rk(**Σ**) = *p*, where **Σ** is the covariance matrix of ***y***. For instance, one condition required for ULS or GLS estimation is that the fourth cumulants of ***y*** must be zero, which is the case, for example, if ***y*** follows a multivariate normal distribution (for this and other conditions, see Browne, 1974). For ML estimation note that (*n* − 1)***S*** ∼ W(**Σ**,*n* − 1) if ***y*** ∼ **N**(***μ***,**Σ**).

Another possibility of estimation for the EFA model is principal axis analysis (PAA). The model of PAA is

$$\text{Z}={\text{FL}}^{\prime }+\text{E},$$

where ***Z*** is a *n* × *p* matrix of standardized test results, ***F*** is a *n* × *p* matrix of factor scores, ***L*** is a *p* × *p* matrix of factor loadings, and ***E*** is a *n* × *p* matrix of error terms. For estimation of ***F*** and ***L*** based on the representation ***Z***′***Z*** = ***R*** = ***LL′*** + ***D*** the principal components transformation is applied. However, the eigenvalue decomposition is not based on ***R***, but is based on the reduced correlation matrix $R_{h}=R-\hat{D},$ where $\hat{D}$ is an estimate for ***D***. An estimate $\hat{D}$ is derived using $h_{j}^{2}=1-v_{j}$ and estimating the communalities *h_j_* (for methods for estimating the communalities, see Harman, 1976).

The assumptions of principal axis analysis are

$$\begin{array}{llll} \text{Z}\;\in {\mathbb{R}}^{n\times p}, & \;\text{L}\;\in {\mathbb{R}}^{p\times p},\;\text{F}\;\in {\mathbb{R}}^{n\times p},\;\text{and}\;\text{E}\;\in {\mathbb{R}}^{n\times p}, & & \\ \text{E}\left(\text{f}\right) & =\text{0}, & & \\ \text{E}\left(\text{e}\right) & =\text{0}, & & \\ \text{cov}\left(\text{e},\text{e}\right) & =\text{E}\left({\text{ee}}^{\prime }\right)=\text{D}=\text{diag}\left\{v_{1},\ldots ,v_{p}\right\}, & & \\ \text{cov}\left(\text{f},\text{e}\right) & =\text{E}\left({\text{fe}}^{\prime }\right)=\text{0}, & & \\ \text{cov}\left(\text{f},\text{f}\right) & =\text{E}\left({\text{ff}}^{\prime }\right)=\text{I}, & & \end{array}$$

and empirical moments of the manifest variables are assumed to exist such that, for any manifest variable *j* = 1, …, *p*, its empirical variance is not zero ($s_{j}^{2}\neq 0$). Moreover, we assume that rk(***Z***) = rk(***R***) = *p* and that the matrices ***Z***, ***F***, ***L***, and ***E*** are interval-scaled (at the least).

### 2.3 General remarks

Two remarks are important before we discuss the assumptions associated with the classical factor models in the next section.

First, it can be shown that ***L*** is unique up to an orthogonal transformation. As different orthogonal transformations may yield different correlation patterns, a specific orthogonal transformation must be taken into account (and fixed) before the estimation accuracies of the factor models can be compared. This is known as “rotational indeterminacy” in the factor analysis approach (e.g., see Maraun, 1996). For more information, the reader is also referred to Footnote 8 and Section 7.

Second, the criterion used to determine the number of factors extracted from the data must be distinguished as well. In practice, not all *k* or *p* but instead $\hat{k}<k$ or *p* factors with the $\hat{k}$ largest eigenvalues are extracted. Various procedures are available to determine $\hat{k}$. Commonly used criteria in educational research are the Kaiser-Guttman criterion (Guttman, 1954; Kaiser and Dickman, 1959), the scree test (Cattell, 1966), and the method of parallel analysis (Horn, 1965).

## 3 Assumptions Associated with the Classical Factor Models

The three models described in the previous section in particular assume interval-scaled data and full rank covariance or correlation matrices for the manifest variables. Typically in the exploratory factor analysis model, the manifest variables ***y*** or the standardized variables ***z*** are assumed to be normally distributed. For the PCA and PAA models, we additionally want to presuppose – for computational reasons – that the variances of the manifest variables are substantially large. The EFA and PAA models assume uncorrelated factor terms and uncorrelated error terms (which can be relaxed in the framework of structural equation models; e.g., Jöreskog, 1966), uncorrelatedness between the error and latent ability variables, and expected values of zero for the errors as well as latent ability variables.

The question now arises whether the assumptions are critical when it comes to educational tests or survey data?^4^

### 3.1 Criterion-referenced tests and PISA questionnaire data

From the perspective of applying these models to data of criterion-referenced tests, the last three of the above mentioned assumptions are less problematic. For a criterion-referenced test, it is important that all items of the test are valid for the investigated content. As such, the usual way of excluding items from the analysis when the covariance or correlation matrices are not of full rank does not work for criterion-referenced tests, because this can reduce content validity of a test. A similar argument applies to the assumption of substantially large variances of the manifest variables. As Klauer (1987) suggested and Sturzbecher et al. (2008) have shown for the driving license test in Germany, the variances of the manifest variables of criterion-referenced tests are seldom high, and in general the data obtained from those tests may lead to extracting too few dimensions. However, for the analysis of criterion-referenced tests, the assumption of interval-scaled data and the assumption of normality of the manifest test and latent ability scores are even more problematic. Data from criterion-referenced tests are rarely interval-scaled – instead the items of criterion-referenced tests are often dichotomous (Klauer, 1987). For criterion-referenced tests, it is plausible to have skewed (non-symmetric) test and ability score distributions, because criterion-referenced tests are constructed to assess whether a desired and excessive teaching goal has been achieved or not. In other words, the tested population is explicitly and intensively trained regarding the evaluated ability, and so it is rather likely that most people will have high values on the manifest test score as well as latent ability (e.g., see the German driving license test; Sturzbecher et al., 2008).

The assumption of interval-scaled data and the normality assumption for the manifest test and latent ability scores may also be crucial for the scaling of cognitive data in PISA (OECD, 2012; Chap. 9 therein). In PISA, the generated students’ scores are plausible values. These are randomly drawn realizations basically from a multivariate normal distribution (as the prior) of latent ability values (person ability is modeled as a random effect, a latent variable), in correspondence to a fitted item response theory model (Adams et al., 1997) giving the estimated parameters of the normal distribution. The mean of the multivariate normal distribution is expressed as linear regression of various direct manifest regressors (e.g., administered test booklet, gender) and indirect “latent” or complex regressors obtained by aggregating over manifest and latent context or background variables (e.g., indicators for economic, social, and cultural status) in a principal component analysis. The component scores used in the scaling model as the indirect “latent” regressors are extracted, in the purely computational sense, to account for approximately 95% of the total variance in all the original variables. The background variables may be categorical or dummy-coded and may not be measured at an interval scale (nor be normally distributed). So as we said before, if one can assume that the distribution of latent background information revealed through questionnaires is skewed, we can expect that the empirical distribution of background information computed by principal component analysis is likely to be biased compared to the true distribution. This is suggested by the results of our simulation study. The bias of the empirical distribution in turn may result in biasing the regression expression for the mean. Therefore, special caution has to be taken regarding possible violations of those assumptions, and a minimum of related sensitivity analyses are required and necessary in order to control for their potential effects.

### 3.2 Historical remarks

The primary aim is to review results of previous studies focusing on the impact of violations of model assumptions. As to our knowledge, such studies did not systematically vary the distributions of the factors (in the case of continuous data as well) and primarily investigated the impact of categorical data (however, not varying the latent distributions for the factors). Reviewing results of previous simulation studies based on continuous indicator variables that have compared different estimation methods (including PCA) and have compared different methods for determining the number of factors, as to our knowledge, would have not constituted reviewing relevant literature focusing primarily on the violations of the assumptions associated with those models.

Literature on classical factor models has in particular investigated violations of the assumption of interval-scaled data. In classical factor analysis, Green (1983) simulated dichotomous data based on the 3PL (three parameter logistic) model (Birnbaum, 1968) and applied PCA and PAA to the data, whereat Cattell’s scree test and Horn’s parallel analysis were used as extraction criteria. Although both methods were applied to the same data, the results regarding the extracted factors obtained from the analyses differed, and the true dimensionality was not detected. In general, the models extracted too many factors. These findings are in line with expectations. Green (1983) used the phi-coefficient Φ as the input data, and according to Ferguson (1941), the maximum value of Φ depends on the difficulty parameters of the items. Dependence of *Φ* on item difficulty can in extreme cases lead to factors being extracted solely due to the difficulties of the items. Roznowski et al. (1991) referred to such factors as difficulty factors.

Carroll (1945) recommended to use the tetrachoric correlation *ρ_tet_* for factor analysis of dichotomous data. The coefficient *ρ_tet_* is an estimate of the dependency between two dichotomous items based on the assumption that the items measure a latent continuous ability – an assumption that corresponds to the factor analysis approach. Although one would expect that *ρ_tet_* leads to less biased results as compared to *Φ*, Collins et al. (1986) were able to show that *Φ* was much better suited to capture the true dimensionality than *ρ_tet_*. In simulations, they compared the two correlation coefficients within the principal component analysis, using a version of the scree test as extraction criterion. The simulated data followed the 2PL model with three latent dimensions, and in addition to item discrimination (moderate, high, very high), the item difficulty and its distribution were varied (easy, moderate, difficult, and extreme difficult item parameters; distributed normal, low frequency, rectangular, and bimodal). The coefficient *ρ_tet_* led to better results when the distribution of item difficulty was rectangular. In all other cases, *Φ* was superior to **ρ*_tet_*. But with neither of the two methods it was possible to detect the true number of factors in more than 45% of the simulated data sets. See Roznowski et al. (1991) for another study illustrating the superiority of the coefficient *Φ* to the coefficient *ρ_tet_*.

Clarification for findings in Green (1983), Collins et al. (1986), and Roznowski et al. (1991) was provided by Weng and Cheng (2005). Weng and Cheng varied the number of items, the factor loadings and difficulties of the items, and sample size. The authors used the parallel analysis extraction method to determine the number of factors. However, the eigenvalues of the correlation matrices were computed using a different algorithm, which in a comparative study proved to be more reliable (Wang, 2001). With this algorithm, *Φ* and *ρ_tet_* performed equally well and misjudged true unidimensionality only when the factor loadings or sample sizes were small, or when the items were easy. This means that it was not the correlation coefficient *per* *se* that led to inadequate estimation of the number of factors but the extraction method that was used.

Muthén (1978, 1983, 1984), Muthén and Christoffersson (1981), Dolan (1994), Gorsuch (1997), Bolt (2005), Maydeu-Olivares (2005), and Wirth and Edwards (2007) present alternative or more sophisticated ways for dealing with categorical variables in factor analysis or structural equation modeling. Muthén (1989), Muthén and Kaplan (1992), and Ferguson and Cox (1993) compared the performances of factor analytic methods under conditions of (manifest) non-normality for the observed indicator variables.

We will add to and extend this literature and investigate in this paper whether the classical factor analysis models can reasonably unveil the factorial structure or properties of the population latent ability distribution in educational test data (e.g., obtained from criterion-referenced tests) when the assumption of normality in the latency may not be justified. None of the studies mentioned above has investigated the “true distribution impact” in these problems.

## 4 Simulation Study

A simulation study is used to evaluate the performances of the classical factor analytic approaches when the latent variables are not normally distributed.

True factorial structures under the exploratory factor analysis model are simulated, that is, the values of *n*, *k*, ***L***, ***f***, and ***e*** are varied.^5^ On the basis of the constructed factorial structures, the matrices of the manifest variables are computed. These matrices are used as input data and analyzed with classical factor analytic methods. The estimates (or computed values) The estimates (or computed values) $\hat{k}$, $\hat{L}$, and $\hat{f}$ (or $\hat{F}$) are then compared to the underlying population values. As criteria for “estimation accuracy” we use the number of extracted factors (as compared to true dimensionality), the skewness of the estimated latent ability distribution, and the discrepancy between estimated and true loading matrix. Shapiro-Wilk tests for normality of the ability estimates are presented and distributions of the estimated and true factor scores are compared as well.

Note that in the simulation study metric scale, not dichotomous, items are analyzed. This can be viewed as a baseline informative for the dichotomous indicator case as well (cf. Section 6). The results of the simulation study can serve as a reference also for situations where violations of normality for latent and manifest variables and metric scale data are present. One may expect the reported results to become worse when, in addition to (latent) non-normality of person ability, data are discretized or item statistics are skewed (manifest non-normality).

### 4.1 Motivation and preliminaries

The present simulation study particularly aims at analyzing and answering such questions as:

- To what extent does the estimation accuracy for factorial structure of the classical factor analysis models depend on the skewness of the population latent ability distribution?
- Are there specific aspects of the factorial structure or latent ability distribution with respect to which the classical factor analysis models are more or less robust in estimation when true ability values are skewed?
- Given a skewed population ability distribution does the estimation accuracy for factorial structure of the classical factor analysis models depend on the extraction criterion applied for determining the number of factors from the data?
- Can person ability scores estimated under classical factor analytic approaches be representative of the true ability distribution or properties thereof when this distribution is skewed?

Mattson (1997)’s method can be used for specifying the parameter settings for the simulation study (cf. Section 4.2). We briefly describe this method (for details, see Mattson, 1997). Assume the standardized manifest variables are expressed as ***z*** = ***Aν***, where ***ν*** is the vector of latent variables and ***A*** is the matrix of model parameters. Moreover, assume that ν = ***T***ω, where ***T*** is a lower triangular square matrix such that each component of ν is a linear combination of at most two components of ω, *E*(***vv′***) = **Σ*_ν_*** = ***TT′***, and ***ω*** is a vector of mutually independent standardized random variables ω*_i_* with finite central moments *μ*_1*i*_, *μ*_2*i*_, *μ*_3*i*_, and *μ*_4*i*_, of order up to four. Then

E(z)=ATE(ω)=0

and

$$E\left({\text{zz}}^{\prime }\right)\left(=\text{A}\Sigma_{\nu }{\text{A}}^{\prime }\right)=\text{AT}E\left({\omega \omega }^{\prime }\right){\text{T}}^{\prime }{\text{A}}^{\prime }={\text{ATT}}^{\prime }{\text{A}}^{\prime }.$$

Or equivalently, $E(z_{i}z_{j})={\gamma^{\prime }}_{i}\gamma_{j}$, where $\gamma_{i}={\left({a^{\prime }}_{i}T\right)}^{\prime }$ and ${a^{\prime }}_{i}$ is the *i*-th row of ***A***. Under these conditions the third and fourth order central moments of *z_i_* are given by

$$\begin{array}{llll} & E\left(z_{i}^{3}\right)=\underset{m}{\sum }\;\gamma_{im}^{3}\mu_{3m}\;\text{and} & & \\ & E\left(z_{i}^{4}\right)=\underset{m}{\sum }\;\gamma_{im}^{4}\mu_{4m}+6\underset{m\geq 2}{\sum }\;\overset{m-1}{\underset{o=1}{\sum }}\;\gamma_{im}^{2}\gamma_{io}^{2}. & & \end{array}$$

Hence the univariate skewness $\sqrt{\beta_{1i}}$ and kurtosis β_2*i*_ of any *z_i_* can be calculated by

$$\sqrt{\beta_{1i}}=\frac{E\left(z_{i}^{3}\right)}{{\left[E\left(z_{i}^{2}\right)\right]}^{3∕2}}\;\text{and}\;\beta_{2i}=\frac{E\left(z_{i}^{4}\right)}{{\left[E\left(z_{i}^{2}\right)\right]}^{2}}.$$

In the simulation study, the exploratory factor analysis model with orthogonal factors (cov( ***f*** , ***f***) = ***I***) and error variables assumed to be uncorrelated and unit normal (with standardized manifest variables) is used as the data generating model. Let ***A***: = (***L***, ***I_p_***) be the concatenated matrix of dimension *p* × (*k* + *p*), where ***I_p_*** is the unit matrix of order *p* × *p*, and let ***v*** : = ( ***f***′, ***e***′)′ be the concatenated vector of length *k* + *p*. Then we have ***z*** = ***Av*** for the simulation factor model. Let ***T***: = ***I***_(*k*+*p*)×(*k*+*p*)_ and ω: = ν, then ***T*** and ***ω*** satisfy the required assumptions afore mentioned. Hence the skewness and kurtosis of any *z_i_* are given by, respectively,

$$\begin{array}{l} \sqrt{\beta_{1i}}=\frac{\sum_{m=1}^{k+p}a_{im}^{3}\mu_{3m}}{{\left[{a^{\prime }}_{i}a_{i}\right]}^{2}}\;and\\ \beta_{2i}=\frac{\sum_{m=1}^{k+p}a_{im}^{4}\mu_{4m}+6\sum_{m=2}^{k+p}\sum_{o=1}^{m-1}a_{im}^{2}a_{io}^{2}}{{\left[{a^{\prime }}_{i}a_{i}\right]}^{2}}. \end{array}$$

Mattson’s method is used to specify such settings for the simulation study as they may be observed in large scale assessment data. The next section describes this in detail.

### 4.2 Design of the simulation study

The number of manifest variables was fixed to *p* = 24 throughout the simulation study. For the number of factors, we used numbers typically found in large scale assessment studies such as the Progress in International Reading Literacy Study (PIRLS, e.g., Mullis et al., 2006) or PISA (e.g., OECD, 2005). According to the assessment framework of PIRLS 2006 the number of dimensions for reading literacy was four, in PISA 2003 the scaling model had seven dimensions. We decided to use a simple loading structure for ***L***, in the sense that every manifest variable was assumed to load on only one factor (within-item unidimensionality) and that each factor was measured by the same number of manifest variables. In reliance on PIRLS and PISA in our simulation study, the numbers of factors were assumed to be four or eight. We assumed that some of the factors were well explained by their indicators while others were not, with upper rows (variables) of the loading matrix generally having higher factor loadings than lower rows (variables). Thus, the loading matrices employed in our study for the four and eight dimensional simulation models were, respectively,

$$\text{L}=\left(\;\begin{array}{cccc} 0.9 & 0 & 0 & 0\\ 0.8 & 0 & 0 & 0\\ 0.7 & 0 & 0 & 0\\ 0.6 & 0 & 0 & 0\\ 0.5 & 0 & 0 & 0\\ 0.4 & 0 & 0 & 0\\ 0 & 0.8 & 0 & 0\\ 0 & 0.7 & 0 & 0\\ 0 & 0.6 & 0 & 0\\ 0 & 0.5 & 0 & 0\\ 0 & 0.4 & 0 & 0\\ 0 & 0.3 & 0 & 0\\ 0 & 0 & 0.6 & 0\\ 0 & 0 & 0.6 & 0\\ 0 & 0 & 0.5 & 0\\ 0 & 0 & 0.4 & 0\\ 0 & 0 & 0.4 & 0\\ 0 & 0 & 0.3 & 0\\ 0 & 0 & 0 & 0.6\\ 0 & 0 & 0 & 0.5\\ 0 & 0 & 0 & 0.5\\ 0 & 0 & 0 & 0.4\\ 0 & 0 & 0 & 0.3\\ 0 & 0 & 0 & 0.3 \end{array}\right)\;\;\;\text{and}\;\;\;\text{L}=\left(\;\begin{array}{cccccccc} 0.9 & 0 & 0 & 0 & 0 & 0 & 0 & 0\\ 0.8 & 0 & 0 & 0 & 0 & 0 & 0 & 0\\ 0.7 & 0 & 0 & 0 & 0 & 0 & 0 & 0\\ 0 & 0.8 & 0 & 0 & 0 & 0 & 0 & 0\\ 0 & 0.8 & 0 & 0 & 0 & 0 & 0 & 0\\ 0 & 0.7 & 0 & 0 & 0 & 0 & 0 & 0\\ 0 & 0 & 0.8 & 0 & 0 & 0 & 0 & 0\\ 0 & 0 & 0.7 & 0 & 0 & 0 & 0 & 0\\ 0 & 0 & 0.6 & 0 & 0 & 0 & 0 & 0\\ 0 & 0 & 0 & 0.7 & 0 & 0 & 0 & 0\\ 0 & 0 & 0 & 0.7 & 0 & 0 & 0 & 0\\ 0 & 0 & 0 & 0.7 & 0 & 0 & 0 & 0\\ 0 & 0 & 0 & 0 & 0.7 & 0 & 0 & 0\\ 0 & 0 & 0 & 0 & 0.6 & 0 & 0 & 0\\ 0 & 0 & 0 & 0 & 0.6 & 0 & 0 & 0\\ 0 & 0 & 0 & 0 & 0 & 0.6 & 0 & 0\\ 0 & 0 & 0 & 0 & 0 & 0.6 & 0 & 0\\ 0 & 0 & 0 & 0 & 0 & 0.5 & 0 & 0\\ 0 & 0 & 0 & 0 & 0 & 0 & 0.5 & 0\\ 0 & 0 & 0 & 0 & 0 & 0 & 0.4 & 0\\ 0 & 0 & 0 & 0 & 0 & 0 & 0.4 & 0\\ 0 & 0 & 0 & 0 & 0 & 0 & 0 & 0.4\\ 0 & 0 & 0 & 0 & 0 & 0 & 0 & 0.4\\ 0 & 0 & 0 & 0 & 0 & 0 & 0 & 0.3 \end{array}\right).$$

**Figure 1:**
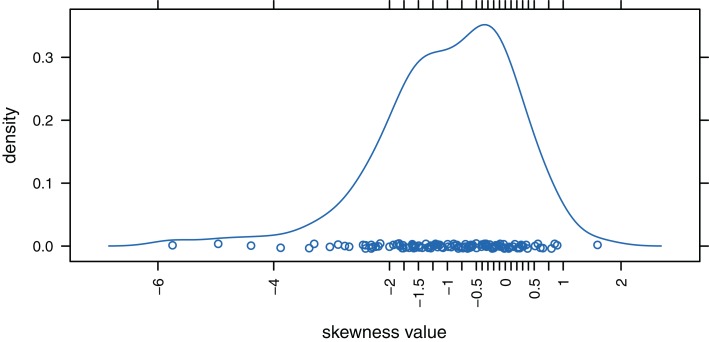
**Distribution of the skewness values for the 125 PIRLS test items**.

We decided to analyze released items of the PIRLS 2006 study (IEA, 2007) to have an empirical basis for the selection of skewness values for ω(= ν). We used a data set of dichotomously scored responses of 7,899 German students to 125 test items. Figure 1 displays the distribution of the PIRLS items’ (empirical) skewness values.^6^

We decided to simulate under three conditions for the distributions of ω. Under the first condition, ω*_m_* (*m* = 1, …, *k*) are normal with *μ*_1*m*_ = 0, *μ*_2*m*_ = 1, *μ*_3*m*_ = 0, and *μ*_4*m*_ = 3. Under the second condition, ω*_m_* (*m* = 1, …, *k*) are slightly skewed with *μ*_1*m*_ = 0, *μ*_2*m*_ = 1, *μ*_3*m*_ = −0.20, and *μ*_4*m*_ = 3. Under the third condition, ω*_m_* (*m* = 1, …, *k*) are strongly skewed with *μ*_1*m*_ = 0, *μ*_2*m*_ = 1, *μ*_3*m*_ = − 2, and *μ*_4*m*_ = 9. The error terms were assumed to be unit normal, that is, we specified *μ*_1*h*_ = 0, *μ*_2*h*_ = 1, *μ*_3*h*_ = 0, and *μ*_4*m*_ = 3 for ω*_h_* (*h* = *k* + 1, …, *k* + *p*). Skewness and kurtosis of any *z_i_* under each of the three conditions were computed using Mattson’s method (Section 4.1). The values are reported in Tables 1 and 2 for the four and eight dimensional factor spaces, respectively.

**Table 1:** **Theoretical values of skewness and kurtosis for *z_i_* (four factors)**.

*z_i_*	Latent variable					
	Normal^a^		Slightly skewed^b^		Strongly skewed^c^	
	$\sqrt{\beta_{1i}}$	β_2*i*_	$\sqrt{\beta_{1i}}$	β_2*i*_	$\sqrt{\beta_{1i}}$	β_2*i*_
*z*_1_	0	3	−0.060	3	−0.599	4.202
*z*_2_	0	3	−0.049	3	−0.488	3.914
*z*_3_	0	3	−0.038	3	−0.377	3.649
*z*_4_	0	3	−0.027	3	−0.272	3.420
*z*_5_	0	3	−0.018	3	−0.179	3.240
*z*_6_	0	3	−0.010	3	−0.102	3.114
*z*_7_	0	3	−0.049	3	−0.488	3.914
*z*_8_	0	3	−0.038	3	−0.377	3.649
*z*_9_	0	3	−0.027	3	−0.272	3.420
*z*_10_	0	3	−0.018	3	−0.179	3.240
*z*_11_	0	3	−0.010	3	−0.102	3.114
*z*_12_	0	3	−0.005	3	−0.047	3.041
*z*_13_	0	3	−0.027	3	−0.272	3.420
*z*_14_	0	3	−0.027	3	−0.272	3.420
*z*_15_	0	3	−0.018	3	−0.179	3.240
*z*_16_	0	3	−0.010	3	−0.102	3.114
*z*_17_	0	3	−0.010	3	−0.102	3.114
*z*_18_	0	3	−0.005	3	−0.047	3.041
*z*_19_	0	3	−0.027	3	−0.272	3.420
*z*_20_	0	3	−0.018	3	−0.179	3.240
*z*_21_	0	3	−0.018	3	−0.179	3.240
*z*_22_	0	3	−0.010	3	−0.102	3.114
*z*_23_	0	3	−0.005	3	−0.047	3.041
*z*_24_	0	3	−0.005	3	−0.047	3.041

*^a^*μ*_1*m*_ = 0, *μ*_2*m*_ = 1, *μ*_3*m*_ = 0, and *μ*_4*m*_ = 3*.

*^b^*μ*_1*m*_ = 0, *μ*_2*m*_ = 1, *μ*_3*m*_ = − 0.20, and *μ*_4*m*_ = 3*.

*^c^*μ*_1*m*_ = 0, *μ*_2*m*_ = 1, *μ*_3*m*_ = − 2, and *μ*_4*m*_ = 9*.

**Table 2:** **Theoretical values of skewness and kurtosis for *z_i_* (eight factors)**.

*z_i_*	Latent variable					
	Normal^a^		Slightly skewed^b^		Strongly skewed^c^	
	$\sqrt{\beta_{1i}}$	β_2*i*_	$\sqrt{\beta_{1i}}$	β_2*i*_	$\sqrt{\beta_{1i}}$	β_2*i*_
*z*_1_	0	3	−0.060	3	−0.599	4.202
*z*_2_	0	3	−0.049	3	−0.488	3.914
*z*_3_	0	3	−0.038	3	−0.377	3.649
*z*_4_	0	3	−0.049	3	−0.488	3.914
*z*_5_	0	3	−0.049	3	−0.488	3.914
*z*_6_	0	3	−0.038	3	−0.377	3.649
*z*_7_	0	3	−0.049	3	−0.488	3.914
*z*_8_	0	3	−0.038	3	−0.377	3.649
*z*_9_	0	3	−0.027	3	−0.272	3.420
*z*_10_	0	3	−0.038	3	−0.377	3.649
*z*_11_	0	3	−0.038	3	−0.377	3.649
*z*_12_	0	3	−0.038	3	−0.377	3.649
*z*_13_	0	3	−0.038	3	−0.377	3.649
*z*_14_	0	3	−0.027	3	−0.272	3.420
*z*_15_	0	3	−0.027	3	−0.272	3.420
*z*_16_	0	3	−0.027	3	−0.272	3.420
*z*_17_	0	3	−0.027	3	−0.272	3.420
*z*_18_	0	3	−0.018	3	−0.179	3.240
*z*_19_	0	3	−0.018	3	−0.179	3.240
*z*_20_	0	3	−0.010	3	−0.102	3.114
*z*_21_	0	3	−0.010	3	−0.102	3.114
*z*_22_	0	3	−0.010	3	−0.102	3.114
*z*_23_	0	3	−0.010	3	−0.102	3.114
*z*_24_	0	3	−0.005	3	−0.047	3.041

*^a^*μ*_1*m*_ = 0, *μ*_2*m*_ = 1, *μ*_3*m*_ = 0, and *μ*_4*m*_ = 3*.

*^b^*μ*_1*m*_ = 0, *μ*_2*m*_ = 1, *μ*_3*m*_ = − 0.20, and *μ*_4*m*_ = 3*.

*^c^*μ*_1*m*_ = 0, *μ*_2*m*_ = 1, *μ*_3*m*_ = − 2, and *μ*_4*m*_ = 9*.

Under the slightly skewed distribution condition, the theoretical values of skewness for the manifest variables range between −0.060 and −0.005, a condition that captured approximately 20% of the considered PIRLS test items. Under the strongly skewed distribution condition, the theoretical values of skewness lie between −0.599 and −0.047, a condition that covered circa 30% of the PIRLS items (cf. Figure 1). Based on these theoretical skewness and kurtosis statistics, we can see to what extent under these model specifications the distributions of the manifest variables deviate from the normal distribution.

How to generate variates ω*_i_* (*i* = 1, …, *k* + *p*) such that they possess predetermined moments *μ*_1*i*_, *μ*_2*i*_, *μ*_3*i*_, and *μ*_4*i*_? To simulate values for ω*_i_* with predetermined moments, we used the generalized lambda distribution (Ramberg et al., 1979)

$$\omega_{i}=\lambda_{1}+\frac{u^{\lambda_{3}}-{\left(1-u\right)}^{\lambda_{4}}}{\lambda_{2}},$$

where *u* is uniform (0, 1), λ_1_ is a location parameter, λ_2_ a scale parameter, and λ_3_ and λ_4_ are shape parameters. To realize the desired distribution conditions for the simulation study (normal, slightly skewed, strongly skewed) using this general distribution its parameters λ_1_, λ_2_, λ_3_, and λ_4_ had to be specified accordingly. Ramberg et al. (1979) tabulate the required values for the λ parameters for different values of *μ*. In particular, for a (more or less) normal distribution with *μ*_1_ = 0, *μ*_2_ = 1, *μ*_3_ = 0, and *μ*_4_ = 3 the corresponding values are λ_1_ = 0, λ_2_ = 0.197, λ_3_ = 0.135, and λ_4_ = 0.135. For a slightly skewed distribution with *μ*_1_ = 0, *μ*_2_ = 1, *μ*_3_ = − 0.20, and *μ*_4_ = 3, the values are λ_1_ = 0.237, λ_2_ = 0.193, λ_3_ = 0.167, and λ_4_ = 0.107. For a strongly skewed distribution with *μ*_1_ = 0, *μ*_2_ = 1, *μ*_3_ = − 2, and *μ*_4_ = 9, the parameter values are given by λ_1_ = 0.993, λ_2_ = − 0.108·10^−2^, λ_3_ = − 0.108·10^−2^, and λ_4_ = − 0.041·10^−3^.

*Remark*. Indeed, various distributions are possible (see Mattson, 1997); however, the generalized lambda distribution proves to be special. It performs very well in comparison to other distributions, when theoretical moments calculated according to the Mattson formulae are compared to their corresponding empirical moments computed from data simulated under a factor model (based on that distribution). For details, see Reinartz et al. (2002). These authors have also studied the effects of the use of different (pseudo) random number generators for realizing the uniform distribution in such a comparison study. Out of three compared random number generators – RANUNI from SAS, URAND from PRELIS, and RANDOM from Mathematica – the generator RANUNI performed relatively well or better. In this paper, we used the SAS program for our simulation study.^7^

Besides the number of factors and the distributions of the latent variables, sample size was varied. In the small sample case, every *z_i_* consisted of *n* = 200 observations, and in the large sample case *z_i_* contained *n* = 600 observations. Table 3 summarizes the design of the simulation study. Overall there are 12 conditions and for every condition 100 data sets were simulated.

**Table 3:** **Summary of the simulation design and number of generated data sets**.

Sample size	Number of factors	Latent variable distribution		
		Normal	Slightly skewed	Strongly skewed
200	4	100	100	100
	8	100	100	100
600	4	100	100	100
	8	100	100	100

Each of the generated 1,200 data sets were analyzed using all of the models of principal component analysis, exploratory factor analysis (ML estimation), and principal axis analysis altogether with a varimax rotation (Kaiser, 1958).^8^ For any data set under each model, the factors, and hence, the numbers of retained factors were determined by applying the following three extraction criteria or approaches: the Kaiser-Guttman criterion, the scree test, and the parallel analysis procedure.^9^

### 4.3 Evaluation criteria

The criteria for evaluating the performance of the classical factor models are the number of extracted factors (as compared to true dimensionality), the skewness of the estimated latent ability distribution, and the discrepancy between the estimated and the true loading matrix. The latter two criteria are computed using the true number of factors. Furthermore, Shapiro-Wilk tests for assessing normality of the ability estimates are presented and distributions of the estimated and true factor scores are compared.

For the skewness criterion, under a factor model and a simulation condition, for any data set the factor scores on a factor were computed and their empirical skewness was the value for this data set that was used and plotted. For the discrepancy criterion, under a factor model and a simulation condition, for any data set *i* = 1, …, 100 a discrepancy measure *D_i_* was calculated,

$$D_{i}=\frac{\sum_{x=1}^{p}\sum_{y=1}^{k}\left|{\hat{l}}_{i;xy}-l_{xy}\right|}{kp},$$

where ${\hat{l}}_{i;xy}$ and *l_xy_* represent the entries of the estimated (varimax rotated, for data set *i* = 1, …, 100) and true loading matrices, respectively. It gives the averaged sum of the absolute differences between the estimated and true factor loadings. We also report the average and variance (or standard deviation) of these discrepancy measures, over all simulated data sets,

$$\bar{D}=\frac{1}{100}\overset{100}{\underset{i=1}{\sum }}\;D_{i}\;\text{and}\;s^{2}=\frac{1}{100-1}\overset{100}{\underset{i=1}{\sum }}\;{\left(D_{i}-\bar{D}\right)}^{2}.$$

In addition to calculating estimated factor score skewness values, we also tested for univariate normality of the estimated factor scores. We used the Shapiro-Wilk test statistic *W* (Shapiro and Wilk, 1965). In comparison to other univariate normality tests, the Shapiro-Wilk test seems to have relatively high power (Seier, 2002). In our study, under a factor model and a simulation condition, for any data set the Shapiro-Wilk test statistic’s *p*-value was calculated for the estimated factor scores on a factor and the distribution of the *p*-values obtained from 100 simulated data sets was plotted.

## 5 Results

We present the results of our simulation study.

### 5.1 Number of extracted factors

**Figure 2:**
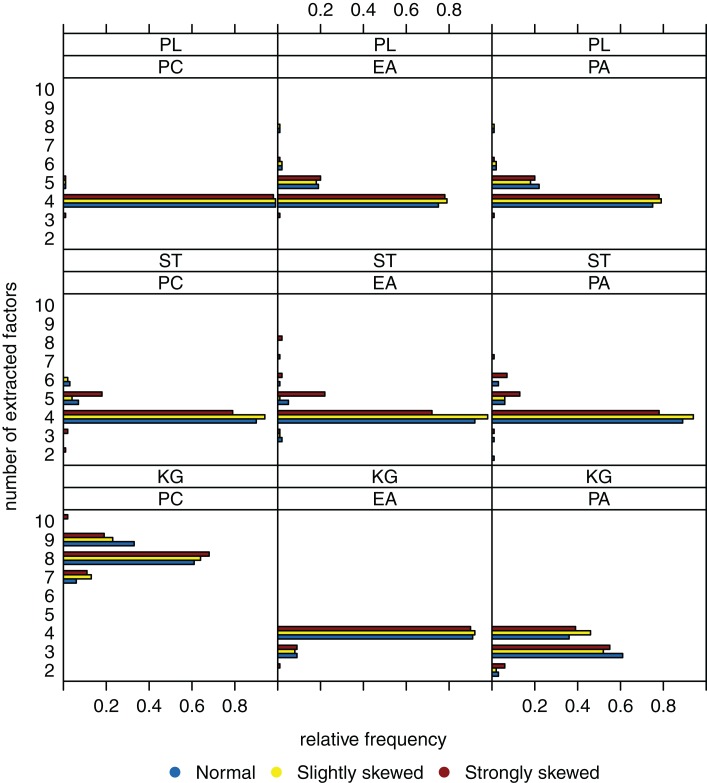
**Relative frequencies of the numbers of extracted factors, for *n* = 200 and *k* = 4**. Factor models are principal component analysis (PCA, or PC), exploratory factor analysis (EFA, or EA), and principal axis analysis (PAA, or PA). Kaiser-Guttman criterion (KG), scree test (ST), and parallel analysis (PL) serve as factor extraction criteria.

Figure 2 shows the relative frequencies of the numbers of extracted factors for sample size *n* = 200 and *k* = 4 as true number of factors. If the Kaiser-Guttman criterion is used, the number of extracted factors is overestimated (for PCA) or tends to be underestimated (for EFA and PAA). With the scree test, four dimensions where extracted in the majority of cases, but variation of the numbers of extracted factors over the different data sets is high. High variation in this case can be explained by the ambiguous and hence difficult to interpret eigenvalue graphics that one needs to visually inspect for the scree test. Applying the parallel analysis method, variation of the numbers of extracted factors can be reduced and the true number of factors is estimated very well (e.g., for PCA). There does not seem to be a relationship between the number of extracted factors and the underlying distribution (normal, slightly skewed, strongly skewed) of the latent ability values.

**Figure 3:**
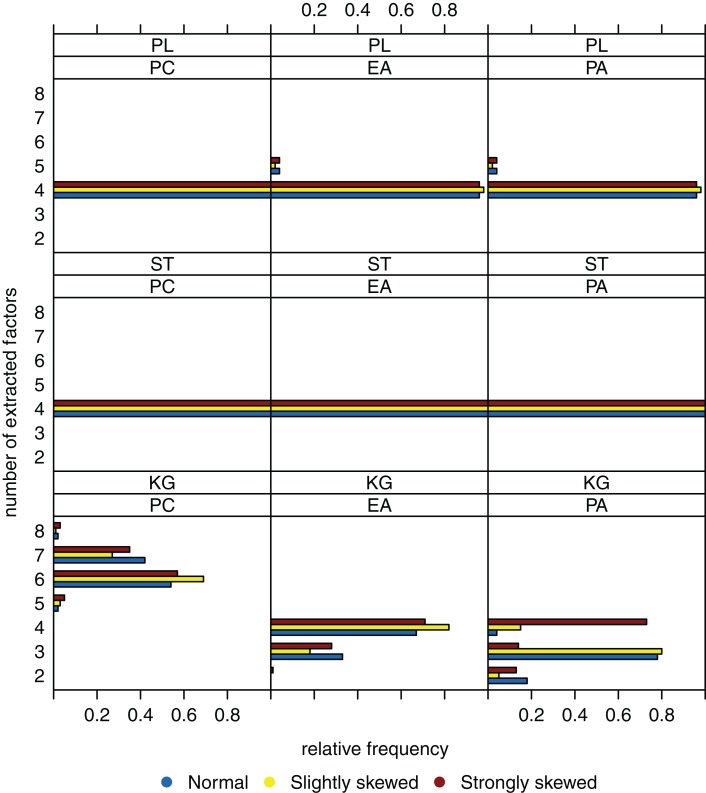
**Relative frequencies of the numbers of extracted factors, for *n* = 600 and *k* = 4**. Factor models are principal component analysis (PCA, or PC), exploratory factor analysis (EFA, or EA), and principal axis analysis (PAA, or PA). Kaiser-Guttman criterion (KG), scree test (ST), and parallel analysis (PL) serve as factor extraction criteria.

When sample size is increased to *n* = 600, variation of the estimated numbers of factors decreases substantially under many conditions (see Figure 3). Compared to small sample sizes, the scree test and the parallel analysis method perform very well. The Kaiser-Guttman criterion still leads to a biased estimation of the true number of factors. Once again, there seems to be no relationship between the distribution of the latent ability values and the number of extracted factors.

**Figure 4:**
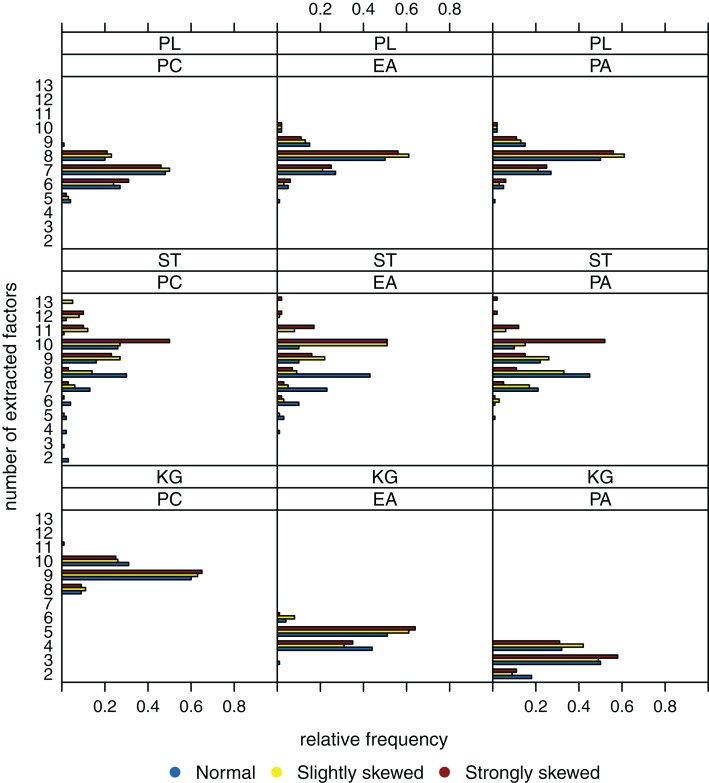
**Relative frequencies of the numbers of extracted factors, for *n* = 200 and *k* = 8**. Factor models are principal component analysis (PCA, or PC), exploratory factor analysis (EFA, or EA), and principal axis analysis (PAA, or PA). Kaiser-Guttman criterion (KG), scree test (ST), and parallel analysis (PL) serve as factor extraction criteria.

Figure 4, for a sample size of *n* = 200, shows the case when there are *k* = 8 factors underlying the data. The Kaiser-Guttman criterion again leads to overestimation or underestimation of the true number of factors. The extraction results for the scree test have very high variation, and estimation of the true number of factors is least biased when the parallel analysis method is used.

**Figure 5:**
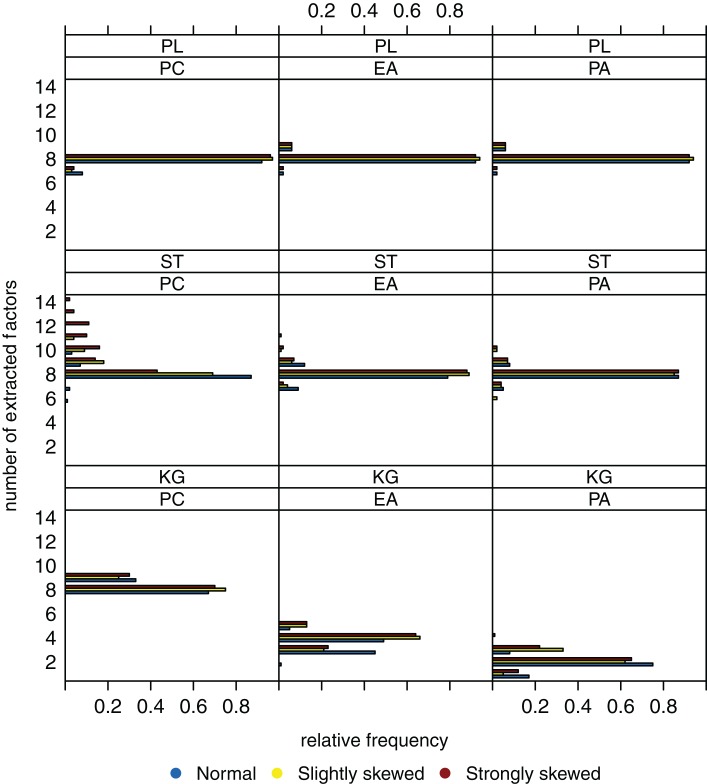
**Relative frequencies of the numbers of extracted factors, for *n* = 600 and *k* = 8**. Factor models are principal component analysis (PCA, or PC), exploratory factor analysis (EFA, or EA), and principal axis analysis (PAA, or PA). Kaiser-Guttman criterion (KG), scree test (ST), and parallel analysis (PL) serve as factor extraction criteria.

Increasing sample size from *n* = 200 to 600 results in a significant reduction of variation (Figure 5). However, the true number of factors can be estimated without bias only when the parallel analysis method is used as extraction criterion. A possible relationship between the distribution of the latent ability values and the number of extracted factors once again does not seem to be apparent.

To sum up, we suppose that the “number of factors extracted” is relatively robust against the extent the latent ability values may be skewed. Another observation is that the parallel analysis method seems to outperform the scree test and the Kaiser-Guttman criterion when it comes to detecting the number of underlying factors.

### 5.2 Skewness of the estimated latent ability distribution

**Figure 6:**
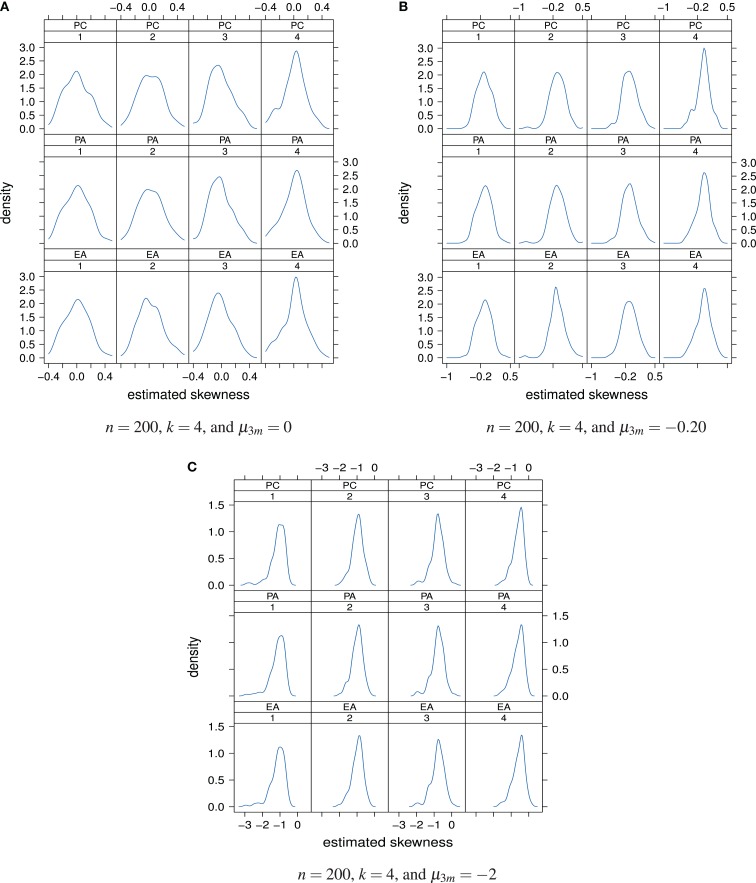
**Distributions of the estimated factor score skewness values as a “function” of factor model and factor position**. Factor models are principal component analysis (PCA, or PC), exploratory factor analysis (EFA, or EA), and principal axis analysis (PAA, or PA). Numbers 1, 2, 3, and 4 stand for 1st, 2nd, 3rd, and 4th factors, respectively. The normal, slightly skewed, and strongly skewed distribution conditions are depicted in the panels **A**, **B**, and **C**, respectively.

Figure 6A shows the distributions of the estimated factor score skewness values, for *n* = 200, *k* = 4, and *μ*_3*m*_ = 0. The majority of the skewness values lies in close vicinity of 0. In other words, for a true normal latent ability distribution with skewness *μ*_3_ = 0, under the classical factor models the estimated latent ability scores most likely seem to have skewness values of approximately 0. An impact of the factor model used for the analysis of the data on the skewness of the estimated latent ability values cannot be seen under this simulation condition. However, the standard deviations of the skewness values clearly decrease from the first to the fourth factor. In other words, the true skewness of the latent ability distribution may be more precisely estimated for the fourth factor than for the first.

When true latent ability values are slightly negative skewed, *μ*_3_ = −0.20, in our simulation study this skewness may only be properly estimated for the first and second extracted factors (Figure 6B). The estimated latent ability values of the third and fourth extracted factors more give skewness values of approximately 0. The true value of skewness for these factors hence may likely to be overestimated.

If true latent ability values are strongly negative skewed, *μ*_3_ = −2, unbiased estimation of true skewness may not be possible (Figure 6C). Even in the case of the first and second factors, the estimation is biased now. True skewness of the latent ability distribution may be overestimated regardless of the used factor model or factor position.

To sum up, under the classical factor models, the concept of “skewness of the estimated latent ability distribution” seems to be sensitive with respect to the extent the latent ability values may be skewed. It seems that, the more the true latent ability values are skewed, the greater is overestimation of true skewness. In other words, strongly negative skewed distributions may not be estimated without bias based on the classical factor models. Increasing sample size, for example from *n* = 200 to 600, or changing the number of underlying factors, say from *k* = 4 to 8, did not alter this observation considerably. For that reason, the corresponding plots at this point of the paper are omitted and can be found in Kasper (2012).

**Figure 7:**
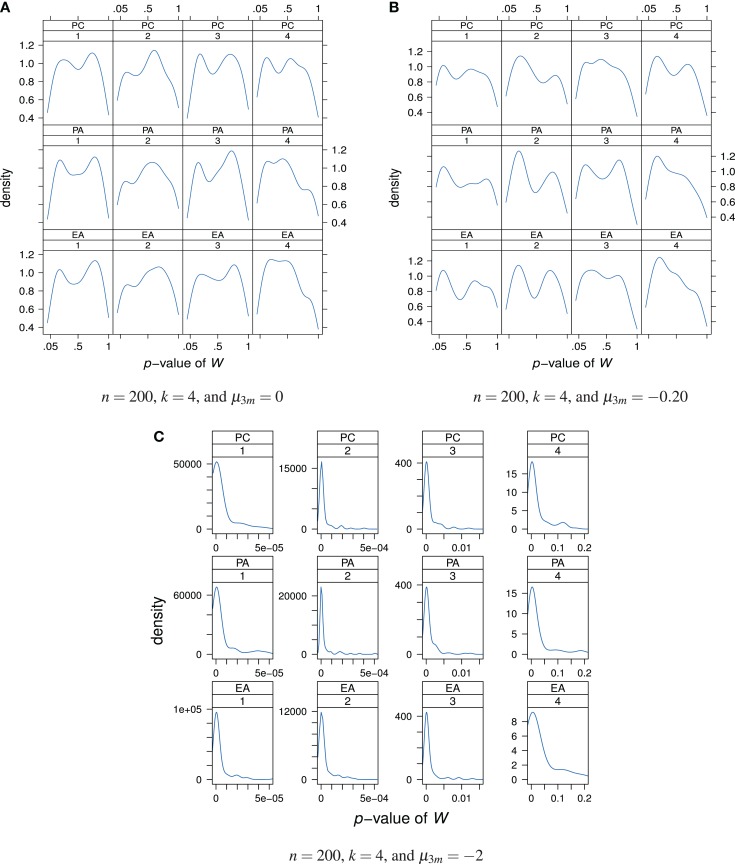
**Distributions of the *p*-values of the Shapiro-Wilk test statistic *W* as a “function” of factor model and factor position**. Factor models are principal component analysis (PCA, or PC), exploratory factor analysis (EFA, or EA), and principal axis analysis (PAA, or PA). Numbers 1, 2, 3, and 4 stand for 1st, 2nd, 3rd, and 4th factors, respectively. The normal, slightly skewed, and strongly skewed distribution conditions are depicted in the panels **A**, **B**, and **C**, respectively.

We performed Shapiro-Wilk tests for univariate normality of the estimated factor scores. As can be seen from Figure 7A, under normally distributed true latent ability scores nearly all values of *W* are statistically non-significant. In these cases, the null hypothesis cannot be rejected.

A similar conclusion can be drawn when the true latent ability values are not normally distributed but instead follow a slightly skewed distribution (Figure 7B). Nearly all Shapiro-Wilk test statistic values are statistically non-significant. In other words, the null hypothesis stating normally distributed latent ability values is seldom rejected although the true latent distribution is skewed and not normal. No relationship between the *p*-values and the used factor model or factor position may be apparent (disregarding the observation that the *p*-values for the fourth factor are generally lower than for the other factors).

The case of a strongly skewed factor score distribution is depicted in Figure 7C. Virtually all values of *W* are statistically significant and the null hypothesis of normality of factor scores is rejected. Similar conclusions or observations may be drawn for increased sample size or factor space dimension and we do omit presenting plots thereof.

**Figure 8:**
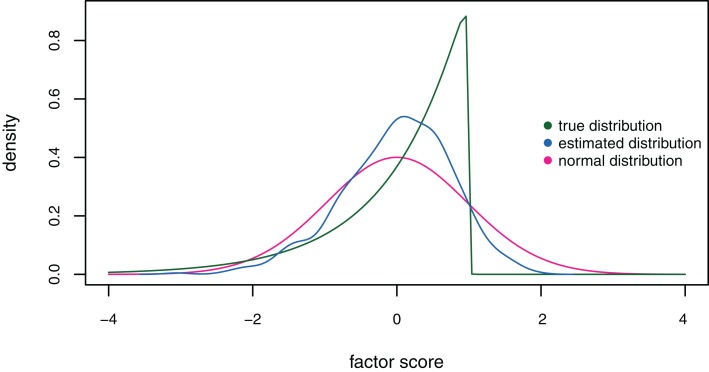
**Distributions of the estimated (blue curve) and true (green curve) factor scores on the fourth factor under the exploratory factor analysis model for sample size *n* = 1,000, factor space dimension *k* = 4, and true skewness *μ*_3_ = − 2**. The unit normal distribution is plotted as a reference (red curve).

Finally, Figure 8 shows the distribution of the estimated factor scores on the fourth factor (for *k* = 4) in comparison to the true strongly skewed ability distribution under the exploratory factor analysis model for a sample size of *n* = 1,000. The unit normal distribution is plotted as a reference. The estimated factor scores have a skewness value of −0.47 compared to true skewness −2. The estimated distribution deviates from the true distribution and does not approximate it acceptably well.

### 5.3 Discrepancy between the estimated and the true loading matrix

In Table 4, the average and standard deviation coefficients $\bar{D}$ and *s* for the discrepancies are reported. The largest average discrepancy values are obtained for the condition *n* = 200, *k* = 8, and the strongly skewed latent ability distribution: 0.173, 0.157, and 0.143 for PCA, EFA, and PAA, respectively. Under this condition, the true factor loadings are, mostly or clearly, overestimated or underestimated. Minor differences between the estimated and true factor loadings are obtained for *n* = 600, *k* = 4, and the normal latent ability distribution: with average discrepancies 0.076, 0.063, and 0.066 for PCA, EFA, and PAA, respectively.

**Table 4:** **Discrepancy averages and standard deviations $\bar{D}$ and *s*, respectively**.

*n*	*k*	Model	Latent variable distribution					
			Normal		Slightly skewed		Strongly skewed	
			$\bar{D}$	*s*	$\bar{D}$	*s*	$\bar{D}$	*s*
200	4	PCA^a^	0.143	0.156	0.143	0.156	0.158	0.173
		EFA^b^	0.129	0.142	0.124	0.136	0.141	0.154
		PAA^c^	0.128	0.139	0.124	0.136	0.137	0.150
600	4	PCA	0.076	0.087	0.075	0.086	0.091	0.106
		EFA	0.063	0.072	0.062	0.072	0.080	0.095
		PAA	0.066	0.075	0.064	0.074	0.082	0.096
200	8	PCA	0.165	0.169	0.162	0.166	0.172	0.176
		EFA	0.154	0.157	0.152	0.155	0.156	0.159
		PAA	0.135	0.138	0.134	0.138	0.143	0.146
600	8	PCA	0.119	0.123	0.118	0.123	0.125	0.130
		EFA	0.106	0.112	0.107	0.112	0.115	0.120
		PAA	0.097	0.101	0.095	0.099	0.102	0.105

*^a^PCA, principal component analysis; ^b^EFA, exploratory factor analysis; ^c^PAA, principal axis analysis*.

**Figure 9:**
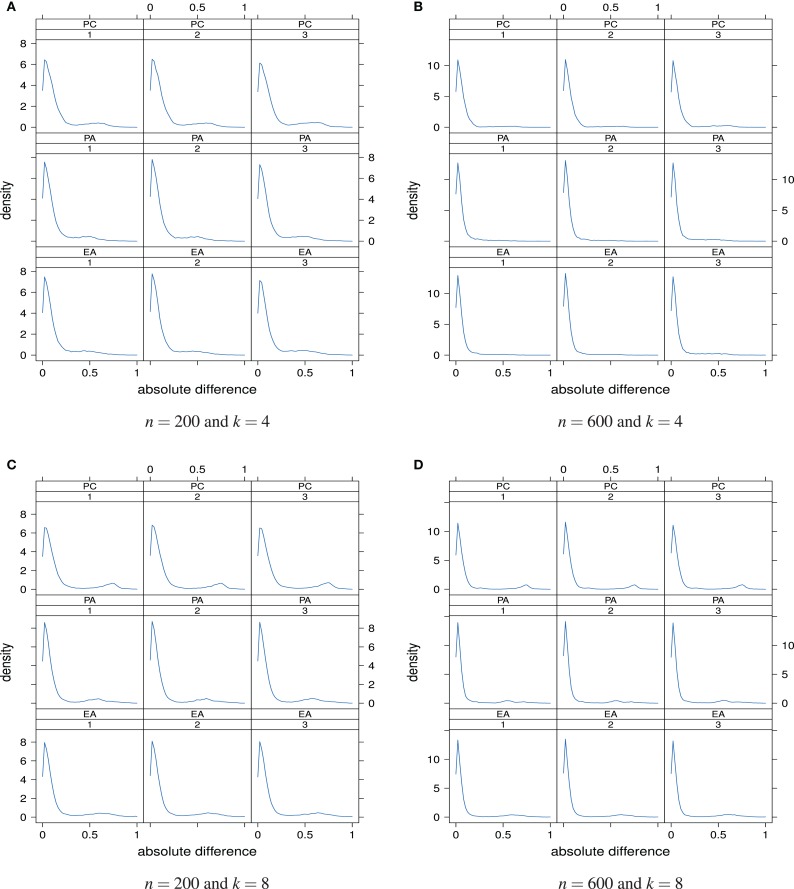
**Distributions of the absolute differences $\left|{\hat{l}}_{i;xy}-l_{xy}\right|$ as a “function” of factor model and skewness of the latent ability distribution**. Factor models are principal component analysis (PCA, or PC), exploratory factor analysis (EFA, or EA), and principal axis analysis (PAA, or PA). Numbers 1, 2, and 3 stand for normal, slightly skewed, and strongly skewed population latent ability values, respectively. The panels are for the different sample sizes and numbers of underlying factors.

Deviations of the estimated loading matrix from the true loading matrix can also be quantified and visualized at the level of individual absolute differences $\left|{\hat{l}}_{i;xy}-l_{xy}\right|$. In this way not only overall discrepancy averages can be studied but also the distribution of absolute differences at the individual entry level. Figure 9 shows the distributions of the absolute differences $\left|{\hat{l}}_{i;xy}-l_{xy}\right|$ for the different sample sizes and numbers of underlying factors. In each panel, 100*pk* absolute differences are plotted.

The majority of the absolute differences lies in the range from 0 to circa 0.20. Larger absolute differences between the estimated and true factor loadings occurred rather rarely. It is also apparent that the 36 distributions hardly differ. This observation suggests that the effects or impacts of sample size, true number of factors, and the latent ability distribution on the accuracy of the classical factor models for estimating the factor loadings are rather weak. In that sense, estimation of the loading matrix seems to be robust overall. In our simulation study, we were not able to see a clear relationship between the distribution of the latent ability values and the discrepancy between the estimated and the true loading matrix.

## 6 Analysis of PIRLS 2006 Data

In addition to the simulation study, the classical factor analytic approaches are also compared on the part of PIRLS 2006 data that we presented in Section 4.2. The booklet design in PIRLS implies that only a selection of the items has been administered to each student, depending on booklet approximately 23–26 test items per student (Mullis et al., 2006). As a consequence, the covariance or correlation matrices required for the factor models can only be computed for the items of a particular test booklet. Since analysis of all thirteen booklets of the PIRLS 2006 study is out of the scope of this paper, we decided to analyze booklet number 4. This booklet contains 23 items, and nine of these items (circa 40% of all items) have skewness values in the range of −0.6 to 0. This skewness range corresponds to the values considered in the simulation study, and no other test booklet had a comparably high percentage of items with skewness values in this range.

Note that in the empirical application dichotomized multi-category items are analyzed. In practice, large scale assessment data are discrete and not continuous. Yet, the metric scale indicator case considered in the simulation study can serve as an informative baseline; for instance (issue of polychoric approximation) to the extent that a product-moment correlation is a valid representation of bivariate relationships among interval-scaled variables (e.g., Flora et al., 2012). In our paper, the simulation results and the results obtained for the empirical large scale assessment application are, more or less, comparable.

In PIRLS 2006, four sorts of items were constructed and used for assigning “plausible values” to students (for details, see Martin et al., 2007). Any item loads on exactly one of the two dimensions “Literacy Experience” (L) and “Acquire and Use Information” (A) and also measures either the dimension “Retrieving and Straightforward Inferencing” (R) or the dimension “Interpreting, Integrating, and Evaluating” (I). Moreover, all of these items are assumed to be indicators for the postulated higher dimension “Overall Reading.” In other words, PIRLS 2006 items may be assumed to be one-dimensional if the “uncorrelated” factor “Overall Reading” is considered (“orthogonal” case), or two-dimensional if any of the four combinations of correlated factors {A, L} × {I, R} is postulated (oblique case). In the latter case, “Overall Reading” may be assumed a higher order dimension common to the four factors. Booklet number 4 covers all these four sorts of PIRLS items.

A total of *n* = 526 students worked on booklet number 4. We investigated these data using principal component analysis, exploratory factor analysis, and principal axis analysis. For determining the number of underlying dimensions, the Kaiser-Guttman criterion, the scree test, and the method of parallel analysis were used. The results of the analyses can be found in Table 5.

**Table 5:** **Number of extracted dimensions for the PIRLS 2006 test booklet number 4, German sample**.

Extraction method	Factor model		
	PCA^a^	EFA^b^	PAA^c^
Kaiser–Guttman criterion	6	6	6
Scree test	1	1	1
Parallel analysis method	1	4	4

*^a^PCA, principal component analysis; ^b^EFA, exploratory factor analysis; ^c^PAA, principal axis analysis*.

The situation at this point is comparable to what we have reported in simulation in Figure 3. The scree test unveils unidimensionality of the test data independent of factor model. The numbers of factors extracted by the parallel analysis method depend on the factor model that was used. For PCA, again as for the scree test, unidimensionality is detected, however for the error component models EFA and PAA, four dimensions are uncovered (see also below). It seems that these “inferential” or “distributional” factor models, to some degree, are sensitive to dependencies among factors. According to the Kaiser-Guttman criterion, which performs worst, there are six dimensions underlying the data for any of the three factor models.

The varimax rotated loading matrices for the exploratory factor analysis and principal axis analysis models with four factors are reported in Tables 6 and 7. Once again, the situation is comparable to what we have obtained in simulation in Table 4 or Figure 9. The estimated loading matrices under EFA and PAA are very similar. Highlighted factor loadings ${\hat{l}}_{xy}\geq 0.30$, for instance, are identically located in the matrices. As can be seen from Tables 6 and 7, substantially different items in regard to their PIRLS contents load on the same factors, and moreover, there are items of same PIRLS contents that show substantial loadings on different factors. We suppose that this may be a consequence of the factors, in this example, most likely being correlated with a postulated common single dimension underlying the factors.

**Table 6:** **Loading matrix for four factors exploratory factor analysis of the PIRLS 2006 data for test booklet number 4, German sample**.

Item	Factor			
	1	2	3	4
R011A01C*^A,R^*	0.15	0.26	**0.39**	−0.05
R011A02M*^A,R^*	0.14	0.28	**0.34**	0.19
R011A03C*^A,R^*	0.16	0.24	0.09	0.03
R011A04C*^A,I^*	**0.39**	0.19	0.10	0.06
R011A05M*^A,R^*	0.22	0.08	0.19	0.21
R011A06M*^A,R^*	0.20	0.03	0.14	0.06
R011A07C*^A,R^*	**0.50**	0.20	0.22	0.15
R011A08C*^A,R^*	**0.35**	0.04	**0.38**	−0.09
R011A09C*^A,I^*	**0.55**	0.18	0.11	0.00
R011A10M*^A,I^*	0.28	0.27	0.22	0.11
R011A11C*^A,I^*	**0.37**	0.06	0.14	0.02
R021E01M*^L,R^*	0.08	**0.45**	0.19	−0.06
R021E02M*^L,R^*	0.02	**0.49**	0.09	0.24
R021E03M*^L,R^*	0.14	**0.34**	−0.02	0.02
R021E04M*^L,R^*	0.17	0.28	0.15	0.02
R021E05C*^L,R^*	0.22	0.23	**0.32**	0.12
R021E06M*^L,R^*	0.17	**0.44**	0.09	0.28
R021E07C*^L,I^*	0.13	0.06	**0.48**	0.22
R021E08M*^L,I^*	**0.32**	0.23	0.04	**0.48**
R021E09C*^L,I^*	**0.45**	0.24	0.02	0.20
R021E10C*^L,I^*	0.27	0.23	0.17	0.07
R021E11M*^L,I^*	0.00	0.01	0.06	**0.40**
R021E12C*^L,I^*	**0.38**	0.17	**0.31**	0.22

*Factor loadings greater or equal 0.30 are highlighted*.

*A, Acquire and Use Information; L, Literary Experience; R, Retrieving and Straightforward Inferencing; I, Interpreting, Integrating, and Evaluating*.

**Table 7:** **Loading matrix for four factors principal axis analysis of the PIRLS 2006 data for test booklet number 4, German sample**.

Item	Factor			
	1	2	3	4
R011A01C*^A,R^*	0.15	0.26	**0.40**	−0.06
R011A02M*^A,R^*	0.14	0.29	**0.33**	0.18
R011A03C*^A,R^*	0.16	0.24	0.09	0.02
R011A04C*^A,I^*	**0.38**	0.20	0.10	0.06
R011A05M*^A,R^*	0.22	0.07	0.19	0.24
R011A06M*^A,R^*	0.19	0.02	0.14	0.07
R011A07C*^A,R^*	**0.50**	0.20	0.22	0.16
R011A08C*^A,R^*	**0.36**	0.03	**0.38**	−0.08
R011A09C*^A,I^*	**0.54**	0.19	0.12	0.00
R011A10M*^A,I^*	0.28	0.27	0.22	0.11
R011A11C*^A,I^*	**0.38**	0.07	0.13	0.02
R021E01M*^L,R^*	0.07	**0.45**	0.19	−0.06
R021E02M*^L,R^*	0.03	**0.49**	0.09	0.24
R021E03M*^L,R^*	0.14	**0.33**	−0.02	0.02
R021E04M*^L,R^*	0.17	0.26	0.16	0.04
R021E05C*^L,R^*	0.21	0.23	**0.32**	0.12
R021E06M*^L,R^*	0.17	**0.44**	0.08	0.27
R021E07C*^L,I^*	0.13	0.06	**0.47**	0.23
R021E08M*^L,I^*	**0.32**	0.24	0.05	**0.46**
R021E09C*^L,I^*	**0.45**	0.24	0.02	0.19
R021E10C*^L,I^*	0.27	0.24	0.17	0.06
R021E11M*^L,I^*	0.00	0.02	0.05	**0.40**
R021E12C*^L,I^*	**0.38**	0.17	**0.30**	0.22

*Factor loadings greater or equal 0.30 are highlighted*.

*A, Acquire and Use Information; L, Literary Experience; R, Retrieving and Straightforward Inferencing; I: Interpreting, Integrating, and Evaluating*.

## 7 Conclusion

### 7.1 Summary

Assessing construct validity of a test in the sense of its factorial structure is important. For example, we have addressed possible implications for the analysis of criterion-referenced tests or for such large scale assessment studies as the PISA or PIRLS. There are a number of latent variable models that may be used to analyze the factorial structure of a test. This paper has focused on the following classical factor analytic approaches: principal component analysis, exploratory factor analysis, and principal axis analysis. We have investigated how accurately the factorial structure of test data can be estimated with these approaches, when assumptions associated with the procedures are not satisfied. We have examined the scope of those methods for estimating properties of the population latent ability distribution, especially when that distribution is slightly or strongly skewed (and not normal).

The estimation accuracy of the classical factor analytic approaches has been investigated in a simulation study. The study has in particular shown that the estimation of the true number of factors and of the underlying factor loadings seems to be relatively robust against a skewed population ability or factor score distribution (see Sections 5.1 and 5.3, respectively). Skewness and distribution of the estimated factor scores, on the other hand, have been seen to be sensitive concerning the properties of the true ability distribution (see Section 5.2). Therefore, the classical factor analytic procedures, even though they are performed with metric scale indicator variables, seem not to be appropriate for estimating properties of ability in the “non-normal case.” Significance of this result on sensitivity of factor score estimation to the nature of the latent distribution has been discussed for the PISA study, which is an international survey with impact on education policy making and the education system in Germany (see Sections 1 and 3.1). In addition to that discussion, the classical factor analytic approaches have been examined in more detail on PIRLS large scale assessment data, corroborating the results that we have obtained from the simulation study (see Section 6).

A primary aim of our work is to develop some basic understanding for how and to what extent the results of classical factor analyses (in the present paper, PCA, EFA, and PAA) may be affected by a non-normal latent factor score distribution. This has to be distinguished from non-normality in the manifest variables, which has been largely studied in the literature on the factor analysis of items (cf. Section 3.2). In this respect, regarding the investigation of non-normal factors, the present paper is novel. However, this is important, since it is not difficult to conceive of the possibility that latent variables may be skewed. Interestingly, moreover we have seen that a purely computational dimensionality reduction method can perform surprisingly well, as compared to the results obtained based on latent variable models. This observation may possibly be coined a general research program: whether genuine statistical approaches (originally based on variables without a measurement error) can work well, perhaps under specific restrictions to be explored, when latent variables are basically postulated, seemingly more closely matching the purpose of analysis.

### 7.2 Outlook

We have discussed possible implications of the findings for criterion-referenced tests and large scale educational assessment. The assumptions of the classical factor models have been seen to be crucial in these application fields. We suggest, for instance, that the presented classical procedures should not be used, unless with special caution if at all, to examine the factorial structure of dichotomously scored criterion-referenced tests. Instead, if model violations of the “sensitive” type are present, better suited or more sophisticated latent variable models can be used (see Skrondal and Rabe-Hesketh, 2004). Examples are item response theory parametric or non-parametric models for categorical response data (e.g., van der Linden and Hambleton, 1997). Furthermore, we would like to mention item response based factor analysis approaches by Bock and Lieberman (1970) or Christoffersson (1975, 1977). We may also pay attention to tetrachoric or polychoric based structural equation models by Muthén (1978, 1983, 1984) and Muthén and Christoffersson 1981).

As with factor analysis a general problem (e.g., Maraun, 1996), we had to deal with the issue of rotational indeterminacy and of selecting a specific rotation. We have decided to use varimax rotation, due to the fact that this rotation is most frequently used in empirical educational studies (for better interpretability of the factors). Future research may cover other rotations (e.g., quartimax or equimax) or the evaluation of parameter estimation by examining the communality estimates for each item (which are not dependent on rotation, but are a function of the factor loadings). Moreover, the orthogonal factor model may not be realistic, as factors are correlated in general. However, in the current study, it may be unlikely that having non-zero population factor loadings for correlated dimensions would substantially affect the findings. In further research, we will have to study the case of the oblique (non-orthogonal) factor model.

The results of this paper provide implications for popular research practices in the empirical educational research field. The methods that we have utilized are traditional and often applied in practice (e.g., by educational scientists), for instance to determine the factorial validity of criterion-referenced tests or to study large scale assessment measurement instruments. In addition, to consider other, more sophisticated fit statistics can be interesting and valuable. For example, such model fit statistics as the root mean square residual, comparative fit index, or the root mean squared error of approximation may be investigated. Albeit these fit statistics are well-known and applied in the confirmatory factor analysis (CFA) context, they could be produced for exploratory factor analysis (given that CFA and EFA are based on the same common factor model).

We conclude with important research questions related to the PISA study. In the context of PISA, principal component analysis is used, in the purely computational sense. Other distributional, inferential, or confirmatory factor models, especially those for the verification of the factorial validity of the PISA context questionnaires, have not been considered. Interesting questions arise: are there other approaches to dimensionality reduction that can perform at least as well as the principal component analysis method in PISA data (e.g., multidimensional scaling; Borg and Groenen, 2005)? Is the 95% extraction rule in principal component analysis of PISA data an “optimal” criterion? How sensitive are PISA results if, for example, the parallel analysis method is used as the extraction criterion? Answering these and other related questions is out of the scope of the present paper and can be pursued in more in-depth future analyses. Nonetheless, the important role of these problems in the PISA context is worth mentioning. The PISA procedure uses not only manifest background information but also principal component scores on complex constructs in order to assign literacy or plausible values to students. Future research is necessary to investigate the effects and possible implications of potentially biased estimates of latent or complex background information on students’ assigned literacy values, and especially, their competence levels, based on which the PISA rankings are reported.

## Conflict of Interest Statement

The authors declare that the research was conducted in the absence of any commercial or financial relationships that could be construed as a potential conflict of interest.

## Footnotes

^1^For the sake of simplicity and for the purpose and analysis of this paper, we want to refer to all of these approaches (PCA, EFA, PAA) collectively as classical factor analysis/analytic methods. Albeit it is known that PCA differs from factor analysis in important aspects, and that PAA rather represents an alternative estimation procedure for EFA. PCA and EFA are different technically and conceptually. PCA seeks to create composite scores of observed variables while EFA assumes latent variables. There is no latent variable in PCA. PCA is not a model and instead is simply a re-expression of variables based on the eigenstructure of their correlation matrix. A statistical model, as is for EFA, is a simplification of observed data that necessarily does not perfectly reproduce the data, leading to the inclusion of an error term. This point is well-established in the methodological literature (e.g., Velicer and Jackson, 1990; Widaman, 2007). Correlation matrix is usually used in EFA, and the models for EFA and PAA are the same. There are several methods to fit EFA such as unweighted least squares (ULS), generalized least squares (GLS), or maximum likelihood (ML). PAA is just one of the various methods to fit EFA. PAA is a method of estimating the model of EFA that does not rely on a discrepancy function such as for ULS, GLS, or ML. This point is made clear, for instance, in MacCallum (2009). In fact, PAA with iterative communality estimation is asymptotically equivalent to ULS estimation. Applied researchers often use PCA in situations where factor analysis more closely matches the purpose of their analysis. This is why we want to include PCA in our present study with latent variables, to examine how well PCA results may approximate a factor analysis model. Such practice is frequently pursued, for example in empirical educational research, as we tried to criticize for the large scale assessment PISA study (e.g., OECD, 2005, 2012). Moreover, the comparison of EFA (based on ML) with PAA in this paper seems to be justified and interesting, as the (manifest) normality assumption in the observed indicator variables for the ML procedure is violated in the simulation study and empirical large scale assessment PIRLS application.

^2^PISA is an international large scale assessment study funded by the Organisation for Economic Co-operation and Development (OECD), which aims to evaluate education systems worldwide by assessing 15-year-old students’ competencies in reading, mathematics, and science. For comprehensive and detailed information, see www.pisa.oecd.org.

^3^For the sake of simplicity and without ambiguity, in this paper we want to refer to component scores from PCA as “factor scores” or “ability values,” albeit components conceptually may not be viewed as latent variables or factors. See also Footnote 1.

^4^Note that, at the latent level, there is no formal assumption that the latent factors (what we synonymously also want to call “person abilities”) are normally distributed. At the manifest level, maximum likelihood estimation (EFA) assumes that the observed variables are normal; ULS and GLS (EFA), PAA (EFA), and PCA do not. The latter two methods only require a non-singular correlation matrix (e.g., see MacCallum, 2009). However, in applications, for example in empirical educational research, one often assumes that the latent ability values follow a normal distribution in the population of reference. Moreover, Mattson (1997)’s method described in Section 4.1 states that there is a connection between the manifest and latent distributions in factor analysis. Hence the question is what implications one can expect if this “implicit assumption” may not be justified. Related to the study and evaluation of the underlying assumptions associated with these classical factor models, this paper, amongst others, shows that the data re-expression method PCA performs surprisingly well if compared to the results obtained based on the latent variable approaches EFA and PAA. Moreover, ML and PAA estimation procedures for EFA are compared with one another, for different degrees of violating the normality assumption at the manifest or latent levels.

^5^Obviously, PCA as introduced in this paper cannot be used as a data generating probability model underlying the population. However, the simulation study shows that PCA results can approximate a factor analysis (cf. also Footnote 1).

^6^All figures of this paper were produced using the R statistical computing environment (R Development Core Team, 2011; www.r-project.org). The source files are freely available from the authors.

^7^For the factor analyses in this paper, we used the SAS program and its PROC FACTOR implementation of the methods PCA, EFA, and PAA. More precisely, variation of the PROC FACTOR statements, run in their default settings, yields the performed procedures PCA, EFA, and PAA (e.g., EFA if METHOD = ML).

^8^Because of rotational indeterminacy in the factor analysis approach (e.g., Maraun, 1996), the results are as much an evaluation of varimax rotation as they are an evaluation of the manipulated variables in the study. For more information, see Section 7.

^9^The Kaiser-Guttman criterion is a poor way to determine the number of factors. However, due to the fact that none of the existing studies has investigated the estimation accuracy of this criterion when the latent ability distribution is skewed, we have decided to include the Kaiser-Guttman criterion in our study. This criterion may also be viewed as a “worst performing” baseline criterion, which other extraction methods need to outperform, as best as possible.
